# Neurophysiological and behavioural correlates of ocrelizumab therapy on manual dexterity in patients with primary progressive multiple sclerosis

**DOI:** 10.1007/s00415-022-11114-x

**Published:** 2022-04-13

**Authors:** Raffaele Dubbioso, Marco Bove, Daniele Boccia, Vincenzo D’Ambrosio, Maria Nolano, Fiore Manganelli, Rosa Iodice

**Affiliations:** 1 Department of Neurosciences, Reproductive Sciences and Odontostomatology, University of Naples Federico II, Via Sergio Pansini, 5. 80131 Napoli, Italy; 2 IRCCS Ospedale Policlinico San Martino, Genova, Italy; 3 Section of Human Physiology, Department of Experimental Medicine, Università Degli Studi Di Genova, 16132 Genoa, Italy; 4 IRCCS Ospedale Policlinico San Martino, Genova, Italy; 5 Department of Neuroscience Genetics, Maternal and Child Health (DINOGMI)Center of Excellence for Biomedical Research (CEBR), University of Genoa, RehabilitationGenoa, Ophthalmology Italy; 6 Department of Advanced Biomedical Sciences, University of Naples Federico II, Naples, Italy; 7 Department of Neurology, Istituti Clinici Scientifici Maugeri IRCCS, 27100 Pavia, Italy

**Keywords:** Upper extremity impairment, Multiple sclerosis, progressive, TMS, Cortical excitability, Disease-modifying therapies

## Abstract

**Background:**

Hand dexterity impairment is a key feature of disability in people with primary progressive multiple sclerosis (PPMS). So far, ocrelizumab, a recombinant humanized monoclonal antibody that selectively depletes CD20-expressing B cells, is the only therapy approved for PPMS and recent analysis reported its ability to reduce the risk of upper limb disability progression. However, the neural mechanisms underlying hand impairment in PPMS and the brain networks behind the effect of ocrelizumab on manual dexterity are not fully understood.

**Objective:**

Main aims of our study were: (i) to investigate neurophysiological and behavioural correlates of hand function impairment in subjects with PPMS, and (ii) to use neurophysiologic and behavioural measures to track the effects of ocrelizumab therapy on manual dexterity.

**Methods:**

Seventeen PPMS patients and 17 healthy-controls underwent routine neurophysiological protocols assessing the integrity of cortico-spinal and somatosensory pathways and advanced transcranial magnetic stimulation (TMS) protocols evaluating inhibitory (short and long interval intracortical inhibition, short-latency afferent inhibition) and facilitatory (motor thresholds, intracortical facilitation, short-interval intracortical facilitation) circuits in the primary motor cortex. All subjects also underwent behavioural analysis of hand dexterity by means of nine-hole peg test and finger movement analysis, and hand strength with handgrip and three-point pinch test. Neurophysiological and clinical assessments of hand functionality were also performed after 1 year of ocrelizumab therapy.

**Results:**

At baseline PPMS patients displayed a significant impairment of hand dexterity and strength compared to healthy controls (all *p* < 0.03). Neurophysiological study disclosed prolonged latencies of standard somatosensory and motor evoked potentials (all *p* < 0.025) and an overall reduction of intracortical excitability at TMS protocols, involving both excitatory and inhibitory circuits. Importantly, hand dexterity impairment, indexed by delayed 9HPT, correlated with TMS protocols investigating cortical sensorimotor integration (short-latency afferent inhibition, SAI), *p* = 0.009. Both parameters, 9HPT (*p* = 0.01) and SAI (*p* = 0.01), displayed a significant improvement after 1 year of therapy with ocrelizumab.

**Conclusion:**

Intracortical sensorimotor networks are involved in hand dexterity dysfunction of PPMS. Ocrelizumab therapy displays a beneficial effect on hand dexterity impairment most likely through intracortical networks implicated in fast sensorimotor integration.

## Introduction

Hand function impairment, caused by sensory, coordination, and motor deficits, is widely reported by patients across all multiple sclerosis (MS) types, although patients with progressive disease may have higher prevalence of hand dysfunction compared with patients with less severe disease [1, 2]. Hand function impairment impacts patients’ ability to perform activities of daily living (ADL), affecting their independence and quality of life [3].

From a clinical point of view, hand function has been largely studied by quantitative testing evaluating the strength by means of handgrip or three-point pinch tests, and the sensorimotor coordination with the nine-hole peg test (9HPT) [4] or dedicated finger movement analysis [5]. From a neurophysiological point of view, parameters such as amplitude and latencies of motor-evoked potentials (MEPs) are commonly used in clinical practice in MS patients to assess corticospinal tract (CST) integrity, and MEPs abnormalities are supposed to be mainly related to strength deficit [6–8].

However, hand function also relies on the fast integration of sensorimotor information that cannot be assessed with standard MEP recordings. Importantly, sensorimotor integration at the cortical level can be probed non-invasively by pairing electrical stimulation of peripheral somatosensory afferents with focal transcranial magnetic stimulation (TMS) targeting the contralateral primary motor cortex (M1), the so-called short latency afferent inhibition (SAI) [9, 10].

Objective quantitative assessment of hand functionality is critical for monitoring overall MS disease progression and evaluating the benefit of MS therapies. So far, ocrelizumab, a recombinant humanized monoclonal antibody that selectively depletes CD20-expressing B cells [11], is the only therapy approved for primary progressive MS (PPMS) [12]. In the Phase III ORATORIO trial in PPMS, ocrelizumab-treated patients had significantly lower rates of clinical and magnetic resonance imaging (MRI)-measured progression as assessed by 12- and 24-week confirmed disability progression on the Expanded Disability Status Scale (EDSS), change in timed 25-foot walk, change in T2-weighted brain lesion volume, and total brain volume loss [12].

The subsequent exploratory analysis showed also that ocrelizumab mitigated the progression of upper extremity impairment using the 9HPT [13], raising the possibility of using the latter parameter for assessing ocrelizumab efficacy in future clinical trials.

Based on this evidence, we first investigated neurophysiological correlates of hand function impairment in subjects with PPMS, evaluating both measures of CST excitability and of sensorimotor integration and correlating them with behavioural data of manual dexterity. We hypothesized that neurophysiological measure of fast sensorimotor integration would be severely affected in PPMS and would be associated with hand dexterity deficit with higher sensitivity than the standard measures of CST excitability. Second, we used neurophysiologic and behavioural measures to track the effects of ocrelizumab therapy on manual dexterity.

## Methods

### Patients

This longitudinal study recruited 17 consecutive PPMS patients eligible to start ocrelizumab therapy according to Italian Drugs Agency (AIFA) criteria.

Inclusion criteria were (1) diagnosis of PPMS at least 12 months before inclusion in the study; (2) absence of clinical or neuroradiological disease activity at brain MRI scan for at least 6 months before assessment; (3) ability to complete behavioural testing (see below). Exclusion criteria were (1) contraindication to transcranial magnetic stimulation (history of epilepsy, recent brain surgery or head trauma, previous stroke, pregnancy, presence of metallic implant, or cardiac pacemaker); (2) treatment with antiepileptic drugs or long-lasting benzodiazepines as they might affect cortical excitability; (3) clinical sensory and cerebellar dysfunction affecting upper limb function, defined as moderate to severe: upper limb dysmetria at the finger-nose test, reduced tactile sensation, reduction in vibration sensation; (4) patients with significant cognitive impairment; (5) EDSS cerebellar functional score > 2.

We also enrolled 17 sex- and age-matched healthy control. The study was approved by the ethical committee of the Federico II University Hospital (N. 100/17) and followed the principles of the Declaration of Helsinki. Written informed consent was obtained from all participants. Finally, all subjects were required to be right-handed as assessed by the Edinburgh handedness inventory.

### Clinical assessment

All patients underwent a clinical evaluation with EDSS assessment and manual dexterity evaluation at baseline (T0) and after four injections of intravenous ocrelizumab (600 mg) over a period of about 12 months (T12).

### Hand function assessment

#### Strength evaluation

All subjects underwent strength evaluation of grip and pinch. Maximal voluntary isometric grip and large three-point strength were measured using a handheld dynamometer (Cit Technics, Haren, Netherlands).

**Fig. 1:**
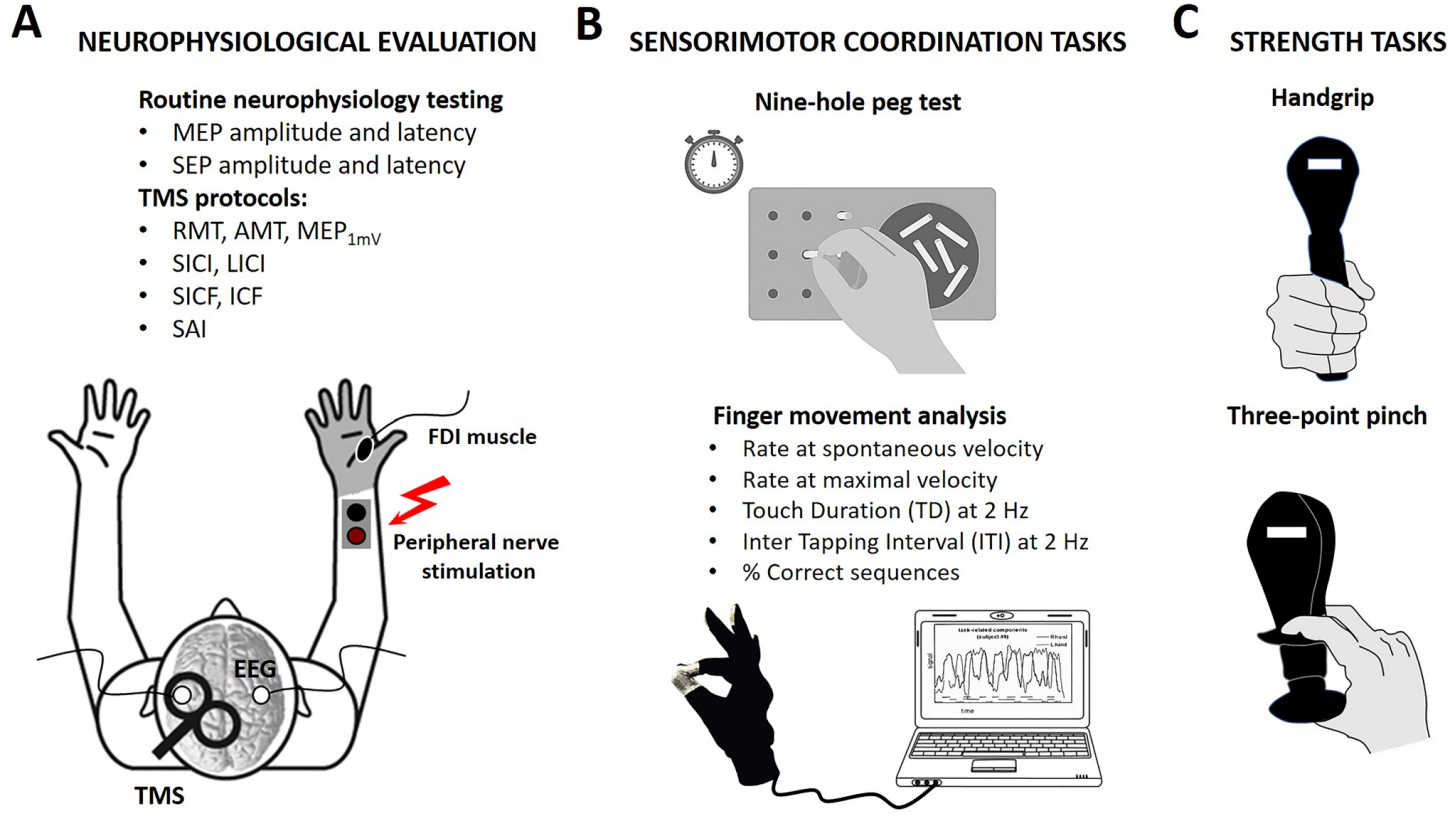
Experimental set-up. **A** Each subject underwent routine neurophysiological assessment evaluating motor (MEP) and somatosensory-evoked potential (SEP) latency and amplitudes, and transcranial magnetic stimulation (TMS) protocols evaluating motor (resting motor threshold, RMT; active motor threshold, AMT; short-interval intracortical inhibition, SICI; long-interval intracortical inhibition, LICI; short-interval intracortical facilitation, SICF; intracortical facilitation, ICF) and sensorimotor cortex (short-latency afferent inhibition, SAI) excitability. Afterward, behavioural protocols were applied to assess the sensorimotor coordination (**B**) and strength aspects (**C**) of hand functionality. **B** Sensorimotor coordination tasks consisted of the 9 Hole-Peg-Test (9HPT) and finger movement analysis with an engineered glove, evaluating five parameters: (i) rate at spontaneous velocity; (ii) rate at maximal velocity; (iii) Touch Duration (TD) at 2 Hz; (iv) Inter Tapping Interval (ITI) at 2 Hz; (v) % Correct sequences. **C** The hand strength was assessed by handgrip and three-point pinch tests

Participants were seated while holding the dynamometer upright, with their elbow bent to approximately 90°, without forearm support. The peak isometric grip and pinch strength (Newton) for two trials was averaged for each hand. In this study, we only considered the dominant hand. See Fig. 1.

#### Sensorimotor coordination tasks

Sensorimotor coordination was assessed at baseline (T0) and after 1 year (T12) by the conventional 9HPT and by the analysis of finger movements with a dedicated engineered glove [5], Fig. 1. As for 9HPT, both hands were tested twice—in two consecutive trials of the dominant hand, followed by two consecutive trials of the nondominant hand—to determine the time taken to complete the test. There was a 300-s time limit per trial. In this study, we only considered the dominant hand.

Finger motor performance was measured by means of a sensor-engineered glove on the dominant hand (GAS, ETT S.p.A., Italy) [5]. All participants were asked to perform self-paced (i.e., right hand at spontaneous velocity), maximal velocity, and metronome-paced (right hand at 2 Hz) repetitive sequences of finger opposition movements (thumb to index-medium-ring-little fingers). Touch Duration (TD) at 2 Hz was computed as the contact time between the thumb and another finger, while Inter Tapping Interval (ITI) at 2 Hz was defined as the time interval between the end of a thumb-to-finger contact and the beginning of the subsequent contact in the finger motor sequence. An eyes-closed paradigm was chosen to exclude possible confounding effects attributable to the integration of acoustic and visual information, and to prevent patients from compensating for possible sensorimotor impairments by visual inspection. Data were acquired at 1 kHz by means of a data acquisition board (USB-1208FS, Measurement Computing, USA). An ad hoc software tool generated the acoustic pacing signal and recorded the occurrence of each tone and of the corresponding finger touch in the motor sequence. Before starting the recording session, all subjects practiced the task at their own spontaneous pace; training ended generally within 2 min when they were able to perform the finger motor sequence without errors. The testing session included three randomly presented 60-s trials (one per condition).

### Electrophysiology

#### Electromyographic (EMG) recording and focal TMS

Participants were seated comfortably in a chair reposing both hands suitably on a cushion or their lap to ensure complete relaxation. Motor-evoked potentials (MEPs) were recorded by electromyography (EMG) from the right first dorsal interosseous (FDI) muscle using Ag–AgCl surface electrodes (AMBU, Ballerup, Denmark) mounted using the belly–tendon technique. The signals from the EMG electrodes were amplified, bandpass filtered (20 Hz–3 kHz), digitized at a frequency of 5 kHz, and stored in a laboratory computer for later offline analysis by Signal software and CED 1401 hardware (Cambridge Electronic Design, Cambridge, UK). The level of baseline EMG activity was controlled by visual feedback through an oscilloscope screen and auditory feedback through a loudspeaker. We rejected trials with involuntary EMG activity from FDI muscle greater than 50 μV in a time window of 500 ms preceding MEPs.

Focal TMS was performed using a figure-of-eight coil (outer diameter of each wing 70 mm) that was held tangentially to the skull with the handle pointing backwards and laterally at an angle of 45° to the sagittal plane (direction of current induced in the brain: posterior to anterior, PA). Experiments were performed by connecting the coil to a high-power magnetic stimulator with a biphasic current waveform (MagPro X100, Medtronic, Denmark). The “hot spot” was defined as the optimal scalp position for eliciting MEPs of maximal amplitude in the contralateral FDI. To ensure stability of the stimulation position over the course of the experiment, the hotspot was marked directly on the scalp with a soft-tip pen.

#### TMS protocols exploring cortical inhibitory and facilitatory networks

Resting motor threshold (RMT) was determined as the minimum stimulator intensity needed to produce a response of at least 50 μV in the relaxed FDI in at least 5 of 10 consecutive trials. Active motor threshold (AMT) was calculated during a mild tonic contraction (approximately 20% of maximal contraction) as the lowest intensity evoking five MEPs of at least 200 μV in ten consecutive trials. MEP_1mV_ was determined as the stimulus intensity, which elicited in the resting FDI an MEP of 1 mV on average in five consecutive trials. At MEP_1mV_ intensity, we then recorded 30 trials to get MEP amplitudes and latencies for each participant. The peak-to-peak amplitude of each MEP was calculated and then averaged. Latency was defined as the shortest latencies identified and measured by visual inspection of superimposed MEP waveforms (Groppa et al. 2012). This measurement was performed by an experienced neurophysiologist (R.I.) who was blinded with respect to the protocol setup.

We also applied ad hoc paired-pulse TMS protocols exploring cortical inhibitory, namely short-interval intracortical inhibition (SICI), long-interval intracortical inhibition (LICI) and short latency afferent inhibition (SAI), and facilitatory circuits such as intracortical facilitation (ICF) and short-interval intracortical facilitation (SICF) (Fig. 1).

SICI and ICF were determined by setting the conditioning stimulus (CS) intensity to 90% AMT and delivering the CS before the test stimulus (TS). For both paradigms, the unconditioned MEP (TS) was adjusted to evoke an MEP of ~1 mV amplitude in the right FDI muscle.

SICI was recorded at interstimulus intervals (ISIs) of 2 and 3 ms, while intracortical ICF was determined at ISIs of 10 and 15 ms [14].

SICF was evaluated as a function of 14 inter-stimulus intervals (ISIs, Fig. 1B), ranging from 1.0 to 3.6 ms with 0.2 ms step, between the first stimulus set to MEP_1mV_ and the second stimulus at 90% [15, 16].

LICI was investigated by implementing 2 suprathreshold stimuli, with the CS adjusted at 120% of the RMT, with ISIs of 100 and 150 ms [17]. The grand mean of SICI, ICF, SICF, and LICI was obtained averaging the ISIs for each protocol.

Finally, SAI was examined at different interstimulus intervals (ISIs) based on the individual N20 wave latency. ISIs ranged from 0 to 8 ms after N20 latency, in steps of 2 ms [18, 19]. Data of patients and controls, obtained at the ISIs 0, 2, 4, 6, and 8, were analyzed and averaged to obtain a grand mean of SAI [20]. The median nerve was stimulated at the wrist through bipolar surface electrodes (cathode proximal, rectangular pulse of 0.2 ms duration). Stimulus intensity was adjusted to produce a slight thumb twitch (120% motor threshold). The intensity of TS was set to MEP_1mV_.

For all paired-pulse paradigms 15 trials were recorded for each condition and randomly intermixed with 15 trials of TS alone (0.2 ± 10% Hz). In addition, the mean peak-to-peak amplitude of the conditioned MEP at each ISI was expressed as a percentage of the mean peak-to-peak amplitude size of the unconditioned test pulse in that block. Complete voluntary muscle relaxation was monitored audio-visually by high-gain EMG (50 µV/division). Trials contaminated with voluntary activity were discarded from the analysis.

#### Somatosensory-evoked potentials (SEPs) of upper limb

SEPs were recorded from scalp silver/silver-chloride (Ag–AgCl) surface electrodes placed at CP3 and the reference electrode at CP4 according to the international 10 to 20 system [21].

The peripheral stimulation was performed over the right median nerve with the anode placed on the wrist crease and the cathode placed 2 cm proximal. Two thousand monophasic square wave pulses of 200 µs duration (Digitimer, Welwyn Garden City, UK) were delivered at a frequency of 3 Hz and at 120% of motor threshold, which is defined as the minimum stimulation intensity able to produce a small twitch of the abductor pollicis brevis muscle. One hundred and twenty milliseconds long EEG trials, starting from 20 ms before the stimulus, were collected at 5 kHz sampling rate by Signal software and CED 1401 hardware (Cambridge Electronic Design, Cambridge, UK). N20 and P25 peak latencies and amplitudes were identified on the average of the trials after band-pass filtering between 1 and 2000 Hz.

### Statistics

Following visual inspection of the data and objective testing using the Kolmogorov–Smirnov test, all data were found to be normally distributed (KS ≥ 0.195, *p* ≥ 0.72), except for EDSS scores (KS $\le$ 0.215, *p* $\le$ 0.04), percentage of correct sequences at the glove system (KS = 0.355, *p* < 0.001), touch duration (KS = 0.163, *p* = 0.022), and 9HPT (KS = 0.266, *p* < 0.001). Normally distributed variables were shown as mean and standard deviation (SD), and differences between HC and PPMS and between patients before and after therapy were analyzed using unpaired t tests and paired t tests, respectively. Non-normally distributed variables were shown as medians and interquartile range (IQR), and Mann–Whitney U tests or Wilcoxon Sign Rank-Sum Test were used to test for differences. Categorical variables were shown as proportions, and the differences were analyzed using *χ*^2^ tests. In addition, at baseline SICF, SAI, SICI, ICF, and LICI (normalized values) were compared with a 2-way mixed model ANOVA, with *ISI* as within-subject factor and *GROUP (HC vs PPMS_t0*) as between subject fact. To test the effects of the therapy, different repeated-measure ANOVAs were performed for each TMS protocol with *THERAPY* (before vs. after treatment) and *ISI* as within-subject factors. If a significant main effect was obtained, group differences were examined with post hoc tests (Bonferroni correction for multiple comparisons). The Greenhouse–Geisser method was used to correct for non-sphericity whenever necessary. The relationship between clinical (dependent) and neurophysiological (independent) variables was explored using regression analysis. The type of regression, the distribution of the residuals, and the presence of outliers and of homoscedasticity were assessed by analysis of relevant scatterplots. Since our main hypothesis was that SAI, a neurophysiological measure of fast cortical sensory–motor integration would predict hand dexterity performances, we applied univariate linear regression modeling to assess the relationship between SAI (independent variable) and clinical measures (dependent variables). Additional regression analyses between the remaining neurophysiological metrics and clinical parameters were applied for a merely explorative purpose. For all the univariate regression models, we reported the *R*-square, the F test for overall significance, and the *p* value. For all statistical analyses, a *p* value < 0.05 was considered significant. SPSS v26.0 (IBM SPSS, NY, USA) was used for the analyses.

## Results

### Participants

Clinical and demographic information are displayed in Table 1. Most of patients (52.9%) displayed a mild level of disability, namely able to walk without aid for 200 m or less (EDSS: 5–5.5). The remaining cohort was constituted of 17.6% of fully ambulatory patients (EDSS: 3–3.5), 11.8% of patients able to walk without aid for 500 m or less (EDSS: 4–4.5), and 17.6% needing walking aids (EDSS: 6–6.5). No patients recruited for this study were undergoing a disease-modifying treatment.

**Table 1:** Clinical, behavioural, and neurophysiological data in Healthy Controls (HC) and Primary Progressive Multiple Sclerosis patients (PPMS) before (t0) and after (t12) ocrelizumab therapy

	HC (*n* = 17)	PPMS_t0 (*n* = 17)	PPMS_t12 (*n* = 17)	*p* value (HC vs PPMS_t0)	*p* value (HC vs PPMS_t12)	*p* value (PPMS_t0 vs PPMS_t12)
Clinical data						
Age	49.88 ± 13.29	50.41 ± 8.38		0.9	0.5	
Sex (M/F)	10/7	8/9		0.5	0.5	
Disease duration (years)	N.A	10.71 ± 6.54		N.A	N.A	
EDSS	N.A	5.5 (1)	5.5 (1)	N.A	N.A	0.32
Behavioural data (dominant side)						
Strength						
Handgrip (Newton)	109.47 ± 31.74	84.24 ± 27.98	94.23 ± 25.7	**0.03**	0.11	0.05
Large three-point pinch (Newton)	113.21 ± 34.62	94.19 ± 25.65	102.2 ± 25.72	0.11	0.26	0.29
Sensory–motor coordination						
Nine-hole peg test (s)	19.09 (3.55)	27.9 (12.78)	24.34 (8.77)	** <0.001**	** <0.001**	**0.01**
Finger movement analysis						
Rate at spontaneous velocity (Hz)	2.34 ± 0.37	1.93 ± 0.39	1.98 ± 0.46	**0.01**	0.08	0.29
Rate at maximal velocity (Hz)	3.25 ± 0.52	2.62 ± 0.5	2.72 ± 0.7	**0.002**	**0.04**	0.21
Touch Duration (TD) at 2 Hz (ms)	192.78 (28.22)	224.56 (62.66)	223.78 (43.8)	**0.003**	**0.009**	0.15
Inter-Tapping Interval (ITI) at 2 Hz (ms)	278.72 ± 59.53	235.53 ± 57.51	246.72 ± 61.6	**0.022**	0.11	0.41
% Correct sequences	97 (0)	93 (0.6)	94 (3)	**0.002**	**0.009**	0.45
Neurophysiological data (dominant side)						
Transcranial magnetic stimulation						
RMT (%)	36.12 ± 8.73	42.82 ± 7.49	40.12 ± 7.98	**0.022**	0.17	0.25
AMT (%)	28.41 ± 8.85	34.47 ± 6.24	30.88 ± 5.45	**0.028**	0.34	0.08
MEP_1mV_ (%)	44.76 ± 12.19	59.41 ± 11.35	59.12 ± 14.12	**0.001**	**0.003**	0.95
MEP amplitude (mV)	1.23 ± 0.94	1.01 ± 0.98	0.97 ± 0.81	0.531	0.35	0.75
MEP latency (ms)	22.14 ± 1.6	25.21 ± 1.95	23.62 ± 5.86	** <0.001**	0.33	0.29
SICI% (2 and 3 ms)	45.93 ± 23.94	59.19 ± 25.64	46.92 ± 25.89	0.12	0.85	0.16
ICF% (10 and 15 ms)	172.57 ± 97.74	161.1 ± 44.98	156.44 ± 40.3	0.6	0.54	0.75
SICF% (1.0 to 3.6 ms with 0.2 ms step)	172.08 ± 72.11	131.04 ± 32.16	128.3 ± 46.34	**0.04**	0.05	0.81
SAI% (0, 2, 4, 6, 8 ms)	70.8 ± 15.65	96.1 ± 15.64	83.15 ± 21	**0.001**	0.06	**0.01**
LICI% (100 and 150 ms)	21.87 ± 18.33	63.43 ± 22.14	72.35 ± 38.34	**0.016**	**0.006**	0.16
Somatosensory-evoked potential						
N20 latency (ms)	19.81 ± 1.04	20.84 ± 1.46	20.62 ± 1.94	**0.025**	0.14	0.7
N20 amplitude (µV)	3.64 ± 1.79	1.48 ± 0.91	1.33 ± 1.11	** <0.001**	** <0.001**	0.62
P25 latency (ms)	24.37 ± 1.41	26.52 ± 3.14	26.36 ± 4.11	**0.017**	0.07	0.9
P25 amplitude (µV)	6.21 ± 2.82	2.62 ± 1.73	2.4 ± 1.82	** <0.001**	** <0.001**	0.65

The table reports the median and inter quartile range (IQR) of EDSS, Nine-hole peg test, Touch duration at 2 Hz, % of corrected sequences and the mean and ± standard deviation of the other variables. Primary progressive multiple sclerosis at baseline (PPMS_t0) and after therapy (PPMS_t12). RMT = resting motor threshold; AMT = active motor threshold; SICI = mean short-interval intracortical inhibition (2 and 3 ms); ICF = mean intracortical facilitation (10 and 15 ms); SICF = mean short interval intracortical facilitation (1, 1.2, 1.4, 1.6, 1.8, 2, 2.2, 2.4, 2.6, 2.8, 3, 3.2, 3.4, 3.6 ms); SAI = mean short-latency afferent inhibition (0, 2, 4, 6, 8 ms); LICI = mean long-interval intracortical inhibition (100 and 150 ms). In bold significant *p* < 0.05

Overall, neurophysiological recordings were well tolerated by both PPMS and controls, and none of the participants reported side effects.

### Behavioural data

At baseline, PPMS patients showed reduced grip strength (*p* = 0.03) but not three-point pinch (*p* = 0.1). As for sensorimotor coordination tasks, patients showed worse performances with increased times to complete the NHPT and an overall finger movement slowing for all tested parameters at glove analysis; see Table 1. Interestingly, after ocrelizumab therapy, hand function parameters, both strength and sensorimotor coordination, showed an overall improvement even if reaching statistical significance only for the time to complete the NHPT (*p* = 0.01) and a trend toward gaining strength at handgrip (*p* = 0.05) (Table 1).

### Neurophysiological data at baseline

At baseline, MEPs in PPMS patients showed longer latencies and higher motor thresholds (RMT, AMT and MEP_1mV_), whereas MEP amplitudes were not significantly different from controls (Table 1).

**Fig. 2:**
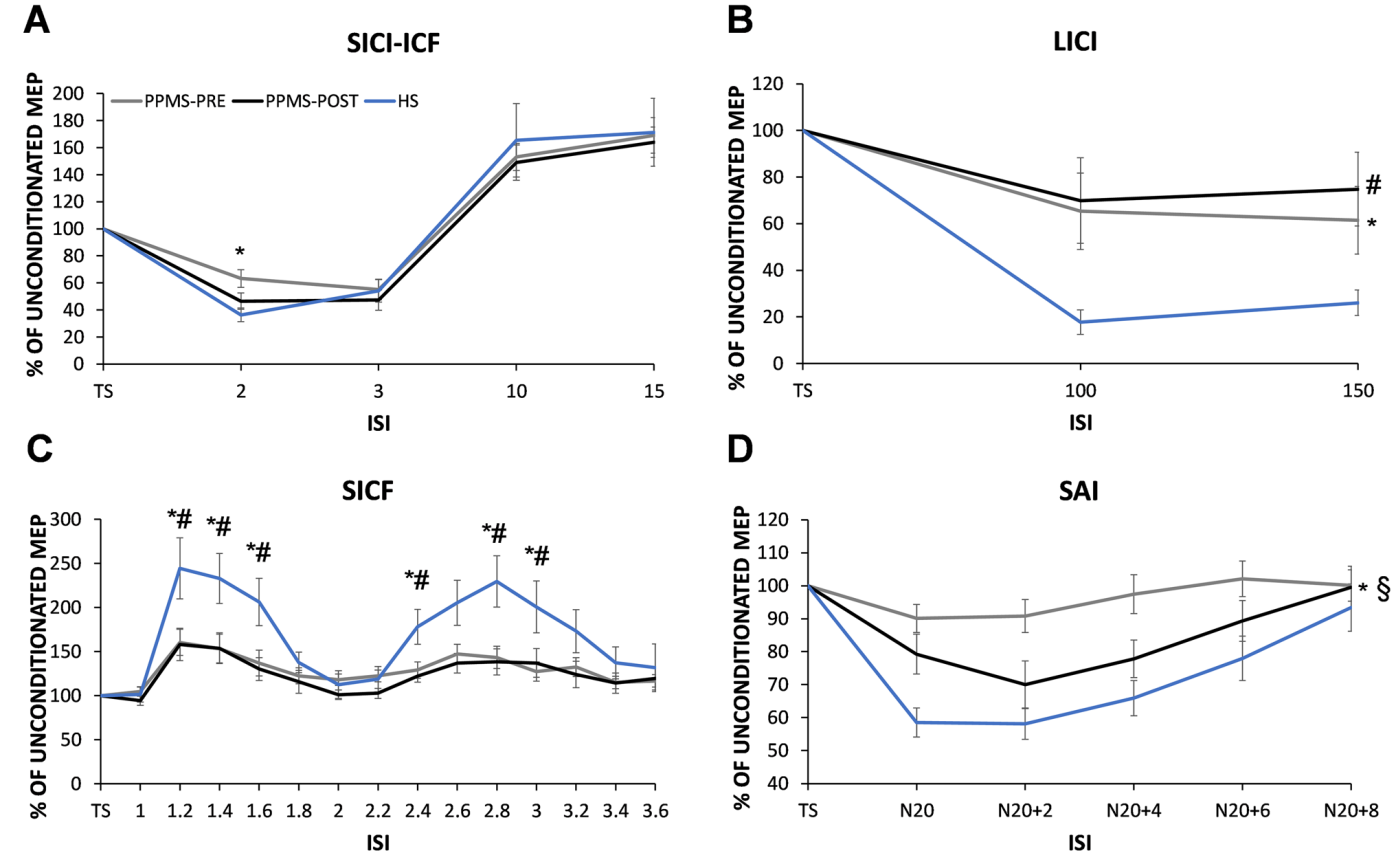
Cortical excitability profiles in healthy subject (HS) and in primary progressive multiple sclerosis patients (PPMS) before (PPMS-PRE) and after (PPMS-POST) ocrelizumab therapy. **A** Group average data normalized with respect to TS for each interstimulus interval (ISI) of short interval intracortical inhibition (SICI) and intracortical facilitation (ICF) showing the lack of inhibition at 2 ms in PPMS patients before therapy (PPMS-PRE, *gray line*) compared to HS (light *blue line*). Conversely after ocrelizumab therapy (PPMS-POST, *black line*), PPMS patients did not show any significant difference compared with HS (**A**). PPMS patients, either pre- or post-treatment, showed an overall altered modulation of cortical inhibition at long intervals (long-interval intracortical inhibition, LICI) and cortical facilitation at short intervals (short-interval intracortical facilitation, SICF) compared with HS (**B**, **C**). **D** Lack of sensorimotor cortical inhibition at short-latency afferent inhibition (SAI) protocol in PPMS patients before therapy, which significantly improved at the end of treatment. MEP = motor-evoked potential; TS = test stimulus. * = HS vs PPMS-PRE; # = HS vs PPMS-POST; § = PPMS-PRE vs PPMS-POST. Significant *p* < 0.05

For SICI, mixed-model ANOVA yielded a *ISI* × *GROUP* effect (*F*_(1,32)_ = 8.637, *p* = 0.006), post-hoc comparisons showed that PPMS patients exhibited an altered modulation for intracortical inhibitory circuits at a specific time point of 2 ms (*p* = 0.002) (Fig. 2A). No significant effect was evident for the ISI (*F*_(1,32)_ = 1.215, *p* = 0.279) and *GROUP* (*F*_(1,32)_ = 2.530, *p* = 0.122) factor.

Alteration of intracortical inhibitory circuits was also confirmed by LICI, showing a main effect of GROUP (*F*_(1,32)_ = 6.995, *p* = 0.013) as indicated by higher values at ISIs 100 and 150 ms in the PPMS cohort (Fig. 2B). On the contrary, the interaction *ISI* × *GROUP* (*F*_(1,32)_ = 1.706, *p* = 0.201) and *ISI* (*F*_(1,32)_ = 0.232, *p* = 0.633) did not reach any statistical significance. TMS protocols exploring motor cortex facilitatory circuits did not show any significant effect for ICF (all *p* > 0.44); on the contrary for SICF, mixed-model ANOVA showed a *ISI *×* GROUP* effect (*F*_(2.75,82.62)_ = 3.350, *p* = 0.026, Greenhouse–Geisser correction: *ε* = 0.212), post hoc comparisons showed that PPMS patients exhibited impaired intracortical facilitatory modulation compared with controls at specific time points of 1.2, 1.4, 1.6, 2.4, 2.8, and 3 (all *p* < 0.047). The overall impairment of SICF in patients was also confirmed by the significant *GROUP* effect (*F*_(1,32)_ = 4.243, *p* = 0.042). As expected, we also showed a main ISI effect (F_(2.15,60.27)_ = 10.901, *p* < 0.001, Greenhouse–Geisser correction: *ε* = 0.212), indicating the modulation of MEP amplitude at specific ISIs (Fig. 2C).

Regarding SEPs, PPMS patients showed significantly longer latencies of N20 and P25 waves associated with lower amplitudes (Table 1). Regarding SAI, statistical analysis yielded a significant *GROUP* effect (*F*_(1,32)_ = 14.466, *p* = 0.001); indeed, PPMS patients exhibited an overall impairment of sensory input inhibition over the motor cortex. As expected, we also showed a main *ISI* effect (*F*_(2.15,60.27)_ = 56.657, *p* < 0.001), being the inhibition gradually less evident for longer ISIs (i.e., N20 + 6 and N20 + 8). No significant effect was evident for the *ISI* × *GROUP* interaction (*F*_(4,128)_ = 1.814, *p* = 0.13); Fig. 2D. Finally, to rule out possible effect of the delay as well as of the reduced magnitude of sensory afferent volley on the between group difference observed for SAI, we ran two additional ANOVA mixed model with SEP latencies (i.e., N20 and P25 latencies) and SEP amplitudes (i.e., N20 and P25 amplitudes) as covariates, and we still observed the significant GROUP effect, *F*_(1,30)_ = 9.537, *p* = 0.004 and *F*_(1,30)_ = 7.276, *p* = 0.011, respectively.

### Association of neurophysiological data and clinical parameters

**Fig. 3:**
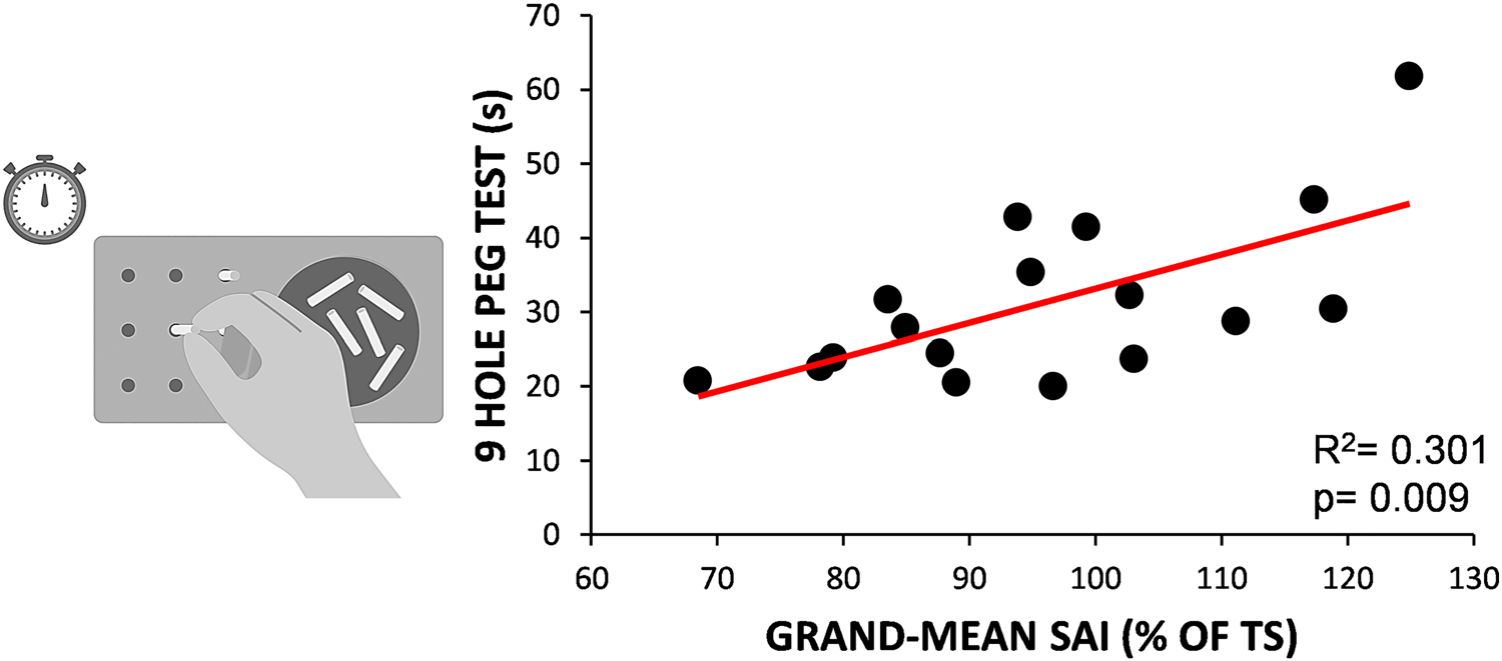
Correlation between short-latency afferent inhibition and the 9-hole peg test. Significant positive correlation between short-latency afferent inhibition (SAI), a neurophysiological measure of intracortical sensorimotor integration, and the 9-hole peg test, a behavioural metric of hand dexterity. Significant *p* < 0.05 at the univariate regression analysis

In patients, the grand mean of SAI predicted the time to complete the 9HPT, with less sensorimotor intracortical inhibition corresponding to greater dexterity impairment (*R*^2^ = 0.301, *F* = 9, *p* = 0.009), Fig. 3. Conversely, absolute SEP latencies and amplitudes were not significantly associated with 9HPT (all *p* > 0.216). The univariate regression analysis did not show any other significant results contrasting SAI and the remaining clinical measures (all *p* > 0.184).

Additional explorative regression analyses disclosed that for gloves parameters, only spontaneous velocity was predicted by the component P25 of SEP (*R*^2^ = 0.314, *F* = 6.866, *p* = 0.019). MEP latencies were correlated with the strength of the dominant upper limb measured with three-point pinch (*R*^2^ = 0.276, *F* = 5.718, *p* = 0.03) and handgrip (*R*^2^ = 0.236, *F* = 4.793, *p* = 0.045), the longer the latencies the weaker the hand. Interestingly, a lower P25 amplitude was also significantly associated with higher EDSS score (*R*^2^ = 0.383, *F* = 9.330, *p* = 0.008) and longer disease duration (*R*^2^ = 0.297, *F* = 6.344, *p* = 0.024). In the healthy control group, univariate regression analysis did not show any significant result contrasting SAI and behavioural data (all *p* > 0.291).

### Changes in neurophysiological data after ocrelizumab therapy

Ocrelizumab therapy did not show any significant effect on motor thresholds (RMT and MEP_1mV_), except for a trend toward lower AMT after treatment (*p* = 0.08, paired t test). The same result was also evident for MEP amplitudes, whereas MEP latencies, even if without reaching statistical significance, were overall shorter than baseline values (Table 1). This result was also confirmed by the direct comparison of controls with patients after therapy (*p* = 0.33, unpaired *t*-test). Paired-pulse protocols, such as SICI, ICF, SICF, and LICI, did not show any significant change over time (Tables 1, 2; Fig. 2). Regarding SAI protocol, ANOVA analysis showed a significant *THERAPY* effect (*F*_(1,16)_ = 7.559, *p* = 0.014) but not *ISI × THERAPY* interaction (*F*_(4,64)_ = 2.363, *p* = 0.062), suggesting that the improvement of the inhibition’s magnitude after treatment was not related to a specific ISI, Fig. 2D. Importantly, we also observed a main *ISI* effect (*F*_(2.75,43.1)_ = 7.049, *p* < 0.001), indicating that the modulation of cortical inhibition across ISIs was maintained over time. No significant effect of therapy was evident for both latency and amplitude of SEPs, which were stable over time (Table 1). This finding suggests that the improvement of SAI was not related to the change of sensory afferent volley but rather to the modification of intracortical sensorimotor circuits.

**Table 2:** ANOVA analysis evaluating the effect of ocrelizumab therapy on paired-pulse TMS protocols

Factor	SICI		ICF		SICF		LICI	
	*F* value	*p* value	*F* value	*p* value	*F* value	*p* value	*F* value	*p* value
*ISI*	2.171	0.16	3.310	0.088	5.487	<0.001	0.006	0.939
*THERAPY*	0.831	0.376	0.107	0.748	0.384	0.545	2.158	0.161
*ISI × THERAPY*	1.064	0.318	0.002	0.961	0.542	0.896	1.780	0.201

*SICI* short-interval intracortical inhibition; *ICF* intracortical facilitation; *SICF* short-interval intracortical facilitation; *LICI* long-interval intracortical facilitation. Significant *p* > 0.05

## Discussion

In this study, we demonstrated that hand dexterity of PPMS patients correlated with the magnitude of SAI, a neurophysiological measure of fast cortical sensorimotor integration. This result suggests that the weaker intracortical sensorimotor pathways, the greater the extent of dexterity impairment. We also found that latency of MEP recorded from upper limb correlated with strength but poorly detected dexterity impairment in patients with MS. In the second part of the study, we used our neurophysiological and behavioural parameters of hand functionality to track the effect of ocrelizumab therapy. Interestingly, we showed that both SAI and 9HPT were the only parameters that exhibited a significant improvement over time.

### Reduced intracortical sensorimotor integration is associated with impaired hand functionality

At baseline PPMS patients showed an overall reduction of cortical excitability, involving both inhibitory and facilitatory circuits. Specifically, we found a reduction of GABA-mediated inhibitory circuits probed by LICI, SICI at 2 ms and SAI and a decrease of facilitatory circuits, indexed by motor thresholds and SICF protocol [22]. The significant decrease of cortical inhibitory [23–26] and facilitatory circuits [25] is in line with the previous results. We also found that patients displayed a significant delay of cortical latency for both somatosensory and motor pathways associated with a reduction of SEP amplitudes.

Among altered neurophysiological parameters, SAI was the best predictor of manual dexterity impairment (i.e., increased 9HPT time), whereas MEP latency was mostly related to strength deficit. In addition, SEP amplitude was the only parameter that significantly correlated with the overall disability and disease duration.

9HPT is considered the optimal metric for measuring the impact of MS on upper extremity function; indeed, it detects progression over time very easily and it is sensitive to treatment [4]. Therefore, finding a neurophysiological correlate of 9HPT is critical to better understand the neural circuits underlying hand dexterity impairment in MS and to find additional biomarkers for the disease. Herein, we demonstrated that SAI, a measure of intracortical sensorimotor integration, was associated with hand performance indexed by 9HPT in PPMS patients. Conversely, 9HPT was not influenced by sensory afferent volley abnormalities; indeed, SEP latency and amplitude did not significantly contribute to the univariate regression model analysis. Interestingly, a recent study of Pisa and colleagues [8] found that hand dexterity impairment in progressive MS was associated with cortico-cortical conduction delay, measured as difference between antero-posterior and lateral-medial stimulation of M1, but not to absolute MEP latency. The importance of cortico-cortical circuits integrity for hand dexterity has been recently demonstrated in healthy controls, where gray matter myelination, measured by the ratio 1/T1 (i.e., R1) at MRI, scaled with hand performance and functional activation during a visuo-motor synchronization task [27]. We can thus speculate that the pathological substrate underpinning cortico-cortical circuits disruption and, therefore, hand performance impairment in MS could be linked to demyelination in the motor cortex and therefore in the cortico-cortical disconnections of the primary motor cortex. However, a pure disruption in the motor network due to neurodegenerative processes cannot be fully ruled out. Only future studies combining high-field MRI with ad hoc TMS protocols could address such important question in MS patients.

Moreover, in line with a previous study [8], we did find an association between absolute MEP latency and hand strength but not with hand dexterity, meaning that standard MEPs can quantify the integrity of the CST but are unable to detect impairment along cortico-cortical networks which are relevant for dexterity performance [27].

### Ocrelizumab therapy improves hand dexterity and sensorimotor integration

We demonstrated that patients after ocrelizumab therapy were clinically and physiologically stable, showing even a slight improvement over time for manual dexterity and SAI magnitude.

These results are in favor of an overall positive effect of ocrelizumab therapy on cortical excitability circuits, mainly on those targeting intracortical sensorimotor integration. Moreover, from a behavioural point of-view, MS patients also displayed an improvement of hand performance at 9HPT after treatment.

Converging evidence supports the hypothesis that SAI is generated in the sensorimotor cortex. Indeed, using invasive recordings of corticospinal volleys in patients with implanted electrodes in the cervical epidural space, the authors showed that peripheral somatosensory input modulates the TMS-induced motor output at the cortical level [18]. This study showed that later I-waves (I2 and I3 waves) but not early I waves were reduced at an interval appropriate for SAI. Therefore, it has been proposed that peripheral nerve stimulation activates glutamatergic thalamocortical projections onto intracortical GABAA-ergic interneurons which, in turn, suppress the intracortical inhibitory GABAA-ergic circuits generating the late descending volleys (late I-waves) in the corticospinal tract [28]. Another study also showed that SAI exhibits a somatotopic organization in the motor cortex, being sensory information able to modulate the excitability of the motor cortex following a center inhibition-surround facilitation profile [10].

Regarding 9HPT, structural MRI studies in progressive MS have showed that worse performance on the 9HPT correlated with cortical gray matter volume atrophy, mainly localized in Brodmann cortical area 44 [29], or alternatively involving a structural brain network including sensory and motor cortices [30].

It is noteworthy that cortical lesions are key features of progressive forms of MS, since they are involved in cognitive impairment and worsening of clinical disability. Interestingly, B cells, the main target of ocrelizumab therapy, are supposed to be involved in cortical pathology by activating a specific cytokine profile able to induce meningeal inflammation and consequently cortical damage [31].

All together, these findings support the hypothesis that the beneficial effect of ocrelizumab treatment on upper limb function seems to act mainly on gray matter alteration rather than on subcortical white matter lesions.

Finally, there are some limitations of our research to be considered. First, in the present study the intensity for evoking MEP_1mV_ was significantly higher in patients compared with controls, raising the possibility that the corticomotoneuronal pool was stimulated closer to saturation in patients, and therefore with less possibility to modulate MEPs amplitudes by conditioning stimuli. Second, the lack of a controlled group treated with placebo could not allow to disentangle the real effect of ocrelizumab therapy on our data. Importantly, in a previous longitudinal study [26], untreated progressive MS patients presented an increase of disability accompanied by a significant decline in cortical excitability of both pyramidal neurons and inhibitory circuits; by contrast, patients receiving immunomodulatory therapy remained stable over time from clinical and neurophysiological point of view; these results suggest that disease-modifying drugs, in our case ocrelizumab, may effectively have a positive impact on disease course. Anyway, further studies are required to confirm our results and the potential value of TMS measurements for follow-up in a larger population of patients with progressive MS.

## Data Availability

The dataset supporting the conclusions of the manuscript will be made available by the authors, to any qualified researcher, without breaching participant confidentiality.
